# Childhood acute leukaemia and residential 16.7 Hz magnetic fields in Germany

**DOI:** 10.1054/bjoc.2000.1630

**Published:** 2001-03

**Authors:** J Schüz, J P Grigat, K Brinkmann, J Michaelis

**Affiliations:** Institut für Medizinische Statistik und Dokumentation der Universität Mainz, D-55101 Mainz, Germany; Forschungsverbund EMV biologischer Systeme, Technische Universität Braunschweig, Postfach 33 29, Braunschweig, D-38023, Germany

**Keywords:** case-control study, child, electromagnetic fields, adverse effects, leukaemia, railroad

## Abstract

We observed a moderate but statistically non-significant association between magnetic fields (MF) and childhood leukaemia. This is the first such study to cover residential exposure to 16.7 Hz (railway frequency) MF though based on few exposed subjects. Our study does not exclude a small excess risk, but the attributable risk must be very low. It is reassuring that neglecting 16.7 Hz MF in childhood cancer studies appears to have little effect on findings. © 2001 Cancer Research Campaign http://www.bjcancer.com


*British Journal of Cancer* (2001) **84**(5), 697–699
© 2001 Cancer Research Campaign
doi: 10.1054/ bjoc.2000.1630, available online at http://www.idealibrary.com on IDEAL

http://www.bjcancer.com

Short Communication

### Childhood acute leukaemia and residential 16.7 Hz magnetic fields in Germany

**J Schüz[1], JP Grigat[2], K Brinkmann[2] and J Michaelis[1]**

[1]Institut für Medizinische Statistik und Dokumentation der Universität Mainz, D-55101 Mainz, Germany; [2]Forschungsverbund EMV biologischer Systeme, Technische Universität Braunschweig, Postfach 33 29, D-38023 Braunschweig, Germany

**Summary** We observed a moderate but statistically non-significant association between magnetic fields (MF) and childhood leukaemia. This is the first such study to cover residential exposure to 16.7 Hz (railway frequency) MF though based on few exposed subjects. Our study does not exclude a small excess risk, but the attributable risk must be very low. It is reassuring that neglecting 16.7 Hz MF in childhood cancer studies appears to have little effect on findings. © 2001 Cancer Research Campaign http://www.bjcancer.com

**Keywords**: case-control study; child; electromagnetic fields, adverse effects; leukaemia; railroad

Extensive epidemiological research has been undertaken to explore the possible association between childhood leukaemia and exposure to residential extremely low-frequency electromagnetic fields (ELF-EMF) (Portier and Wolfe, 1998). Many of those studies assessed the child's exposure by conducting stationary magnetic field measurements over 24 to 48 hours in the child's bedroom (London et al, 1991; Linet et al, 1997; Dockerty et al, 1998; Michaelis et al, 1998; McBride et al, 1999; UKCCSI, 1999; Schüz et al, in press). By doing this, the magnetic field intensity was recorded irrespective of its origin. Thus, magnetic fields produced by power lines as well as by buried wires, indoor wiring or electrical appliances were all taken into account. But since in these studies magnetic fields were measured at power frequency (50/60 Hz), sometimes including higher harmonics, magnetic fields at lower frequencies were neglected. In some European countries, railroad traffic operates at 16.7 Hz (or more precisely $16\frac{2}{3}$ Hz), so elevated magnetic fields may occur in residences close to railroads. In a recent German case-control study on childhood leukaemia and EMF we also measured magnetic fields at 16.7 Hz, and here we present the results. No previous cancer study has covered such exposures at an individual level. A British ecological study on childhood cancer deaths (Knox and Gilman, 1997) observed a significant excess within 4 km of a rail line for all types of cancer combined. But this association cannot be attributed to magnetic field exposure: many other factors may play a role for example, that railroads often cross heavy industrial areas; relevant magnetic field exposures occur only very close to railroads (Schüz et al, 2000). All rail lines were included in the present study, no matter whether electrified or not. Studies in Switzerland (Balli-Antunes et al, 1990), Sweden (Floderus et al, 1993; Alfredsson et al, 1996), Denmark (Guenel et al, 1993) and Norway (Tynes et al, 1994) examined the cancer incidence or mortality among railway workers, but the number of exposed cases was small and the evidence for an increased leukaemia risk among engine drivers or conductors was inconsistent. While in these occupational studies the magnetic field exposure was comparably high (an average of about 20 μT was reported by Tynes et al (1994) but intermittent, the young children in our study were exposed to much lower magnetic field levels (Schüz et al, 2000), but this exposure was more or less continuous.

The methods used in this German study on childhood leukaemia and residential magnetic fields (EMF-study) have been described in detail elsewhere (Schüz et al, 2001). In brief, children diagnosed with acute leukaemia between January 1, 1990 and September 30, 1994 were selected from two recent German case-control studies on childhood cancer and a variety of potential risk factors (Kaatsch et al, 1998; Schüz et al, 1999). Controls were sampled from complete files of population registration offices. For the EMF study, a frequency matched design was adopted (Brookmeyer et al, 1986), which means that all eligible leukaemia cases and all controls of the original case-control studies in the respective diagnostic period were included. In the risk analysis, in which the odds ratio was obtained from a logistic regression model, all subjects were stratified according year of birth, age (age groups of one year) and gender. In addition, in all analyses we adjusted for degree of urbanization (urban, mixed, rural), study origin and socioeconomic status (high, other), which was estimated by parental educational level and monthly family net income.

Measurements over 24 hours were conducted in the child's bedroom of the residence where the child lived longest before the date of diagnosis. We used the FW2a field meter that is based on search coils which record the magnetic field strength in 3 dimensions (Physical Systems Inc, Bradenton, Florida). This measurement instrument enabled us to record the magnetic field intensity specifically at both 50 Hz and at 16.7 Hz. Following the commonly used exposure definition in the ELF-EMF range (Portier and Wolfe, 1998), we used cut-off points of 0.1 μT and 0.2 μT to discriminate between exposure levels.

Measurements at 16.7 Hz were made for 489 cases and 1240 controls. According to the eligibility criteria, the EMF study population comprised 847 cases and 2127 controls so that the response

*Received 2 October 2000*
*Revised 16 November 2000*
*Accepted 16 November 2000*

*Correspondence to:* J Schüz rate for this analysis was 58% among both cases and controls. Major reasons for nonparticipation were refusals, losses of follow up and that the relevant building was pulled down. Due to the availability of measurement instruments, for 25 case and 61 control families only magnetic field measurements at 50 Hz were conducted. Cases were similar to controls with respect to gender, degree of urbanization and residential mobility. The fraction of children aged 4 or less was 57% among cases and 49% among controls ($\chi^2$ *P* value < 0.01). This was due to our study design, since all controls were eligible, even if originally sampled for children with solid tumours, but we controlled for age in the logistic regression model. More parents of controls had a high socioeconomic status than did parents of cases (32% cf. 27%, $\chi^2$ *P* value = 0.04). Hence, all analyses were adjusted for that factor. Despite the strong association with family net income, case or control status was not associated with the type of residence ($\chi^2$ *P* value = 0.26).

Table 1 gives the results for the different exposure measures. Due to the small numbers of exposed subjects (only 0.5% of controls had median magnetic fields ≥ 0.2 μT), the odds ratios lack precision. The odds ratios were increased for median magnetic fields ≥ 0.2 μT (over 24 hours as well as during the night), but not for the intermediate category. No association was seen with peak fields. In an alternative approach we conducted risk analyses based on the distribution of the measurements, but these revealed odds ratios close to one. This was not unexpected, because even cut-off points based on the 95th percentile of all measurements were rather low (e.g., 0.044 μT for median magnetic fields). We could not identify any potential confounding factor that had a more than small effect on the odds ratios (data not shown).

Magnetic field exposure at 50 Hz and 16.7 Hz were combined in two ways. Firstly, children were considered as exposed if the magnetic field exceeded 0.2 μT at least one frequency (there was only one case and no control for which both 50 Hz-median and 16.7 Hz-median were ≥ 0.2 μT). The risk estimates reported for magnetic field exposure at 50 Hz-alone were not substantially altered (Schüz et al, 2001); the odds ratio for 16.7/50 Hz-median magnetic fields decreased slightly to 1.53 (95% confidence interval (CI), 0.71–3.33) compared to 1.55 at 50 Hz. In a second approach, we divided the magnetic field intensities at both frequencies by its corresponding reference levels of 100 μT at 50 Hz and 300 μT at 16.7 Hz, as defined in the German guidelines for exposure of the general public (as recommended by ICNIRP, 1998), and then added the two respective fractions. We defined cut-off points at 1/500 and 1/1000 of the guidelines. The results are given in Table 2. The differences compared to the odds ratios based only on the 50 Hz-magnetic fields were rather small.

Our study is the first to explore the possible association between childhood leukaemia and 16.7 Hz magnetic fields produced by

**Table 1** Risk of childhood leukaemia associated with exposure to residential 16.7 Hz magnetic fields

| | **<0.1 μT** | **0.1 – <0.2 μT** | **≥ 0.2 μT** |
|---|---|---|---|
| Median magnetic field | | | |
| Cases/controls | 484/1216 | 2/18 | 3/6 |
| Odds ratio (95%-CI)[c] | 1.00 | 0.31 (0.07–1.38) | 1.91 (0.41–8.89) |
| Magnetic field at night[a] | | | |
| Cases/controls | 485/1222 | 2/15 | 2/3 |
| Odds ratio (95%-CI)[c] | 1.00 | 0.42 (0.09–1.94) | 1.71 (0.23–12.5) |
| Peak magnetic field[b] | | | |
| Cases/controls | 470/1167 | 11/41 | 8/32 |
| Odds ratio (95%-CI)[c] | 1.00 | 0.62 (0.31–1.25) | 0.71 (0.31–1.60) |

[a]Median magnetic field between 10 pm and 6 am. [b]95th percentile of the individual measurement.
[c]Odds ratio from logistic regression analysis stratified by age, gender, and year of birth and adjusted for socioeconomic status, study origin and degree of urbanization.

**Table 2** Risk of childhood leukaemia associated with exposure to both 16.7 Hz and 50 Hz magnetic fields

| | **<1/1000** | **1/1000 – <1/500** | **≥ 1/500[b]** |
|---|---|---|---|
| Median magnetic field | | | |
| Cases/controls | 441/1138 | 39/82 | 9/20 |
| Odds ratio (95%-CI)[c] | 1.00 | 1.33 (0.87–2.03) | 1.39 (0.59–3.27) |
| Cases/controls | 472/1210 | 33/73 | 9/18 |
| Odds ratio (95%-CI)[c] | 1.00 | 1.15 (0.73–1.81) | 1.56 (0.66–3.70) |
| Magnetic field at night[a] | | | |
| Cases/controls | 444/1158 | 34/71 | 11/11 |
| Odds ratio (95%-CI)[c] | 1.00 | 1.42 (0.90–2.23) | 3.18 (1.25–8.12) |
| Cases/controls | 468/1219 | 34/70 | 12/12 |
| Odds ratio (95%-CI)[c] | 1.00 | 1.42 (0.90–2.23) | 3.28 (1.35–7.95) |

[a]Median magnetic field between 10 pm and 6 am. [b]Expressed in fractions below the limits defined in the German guidelines (100 μT at 50 Hz, 300 μT at 16.7 Hz). [c]Odds ratio from logistic regression analysis stratified by age, gender, and year of birth and adjusted for socioeconomic status, study origin, and degree of urbanization.

© 2001 Cancer Research Campaign railroad traffic. The population basis of our study and the large study population allow a precise estimation of the distribution of 16.7 Hz magnetic fields in German residences (Schüz et al, 2000), but since magnetic fields above 0.2 μT were very rare, the statistical uncertainty in our risk estimates is substantial. The limitations due to this statistical uncertainty and to various potential sources of bias are discussed in detail elsewhere (Schüz et al, 2001). But there are 3 additional issues that should be considered when interpreting the results of the 16.7 Hz magnetic fields analysis.

Firstly, the time lag between the date of diagnosis and the measurement date in our study was between 3 and 10 years. We cannot rule out that in some cases the magnetic field intensity changed materially over time. However, our risk estimates are based on median magnetic fields, which means that magnetic fields exceeded the cut-off point for half a day. These are exceptional situations that do not usually occur close to railroads but only close to extremely busy railroads, train stations, or 16.7 Hz high-voltage power lines. Secondly, we used a cut-off point of 0.2 μT, which was commonly used by previous studies on adverse effects of 50 Hz magnetic fields; 0.2 μT, equals 1/500 of the reference level of the German guidelines (ICNIRP, 1998). The respective proportion of 1/500 of the reference level (300 μT) at 16.7 Hz is 0.6 μT, but the magnetic field exceeded this value in only one residence. Thirdly, there is no known convincing biological mechanism for cancer induction or promotion by ELF-EMF. However, a significant melatonin reduction among Swiss railway engine drivers has been reported (Pfluger and Minder, 1996), while melatonin may play a role in carcinogenesis as an antioxidant or radical scavenger (Reiter, 1997).

Because of the small number of exposed children, our study neither provides noticeable evidence for an association between childhood leukaemia and exposure to 16.7 Hz magnetic fields, nor can a small increase in risk be excluded. Even if there is an increase in risk in the order of 1.5 to 2, as shown by recent meta-analyses on the association of childhood leukaemia with elevated 50 Hz magnetic fields (Ahlbom et al, 2000; Greenland et al, 2000), the attributable risk from 16.7 Hz magnetic fields would be very low (<1 case per year under German conditions (Schüz et al, 2000)). Thus, there are no implications from this study in terms of public health. It is reassuring, however, that after including exposure to 16.7 Hz magnetic fields in our risk analysis, the results for 50 Hz magnetic fields were only marginally altered. Therefore, neglecting magnetic fields produced by railroad traffic in childhood cancer studies on ELF-EMF appears to have only little effect on the overall outcome.

### ACKNOWLEDGEMENTS

This study was funded by the German Ministry for the Environment, Nuclear Safety and Nature Preservation. The authors like to thank Bernd Störmer and Andreas Rüther (Braunschweig), Dr Peter Kaatsch, Dr Uwe Kaletsch and Dr Rolf Meinert (Mainz), and especially thank all families participating in this study.

### REFERENCES

Ahlbom A, Day NE, Feychting M, Roman E, Skinner J, Dockerty JD, Green L, Linet MS, McBride ML, Michaelis J, Olsen J, Tynes T and Verkasalo P (2000) A pooled analysis of magnetic fields and childhood leukemia. *Br J Cancer* **83**: 692–698

Alfredsson L, Hammar N and Karlehagen S (1996) Cancer incidence among male railway engine-drivers and conductors in Sweden, 1976–90. *Cancer Causes Control* **7**: 377–381

Balli-Antunes M, Pfluger DH and Minder CE (1990) The mortality from malignancies of haemotopoietic and lymphocytic systems among railway engine drivers. *Environmetrics* **1**: 121–130

Brookmeyer R, Liang KY and Linet M (1986) Matched case-control designs and overmatched analyses. *Am J Epidemiol* **124**: 693–701

Dockerty JD, Elwood JM, Skegg DCG and Herbison GP (1998) Electromagnetic field exposures and childhood cancers in New Zealand. *Cancer Causes Control* **9**: 299–300

Floderus B, Törnqvist S and Stenlund C (1993) Incidence of selected cancers in Swedish railway workers, 1961–79. *Cancer Causes Control* **5**: 189–194

Greenland S, Sheppard AR, Kaune WT, Poole C and Kelsh MA (2000) A pooled analysis of magnetic fields, wire codes, and childhood leukemia. *Epidemiology* **11**: 624–634

Guenel P, Rasmark P, Bach-Andersen J and Lynge E (1993) Incidence of cancer in persons with occupational exposures to electromagnetic fields in Denmark. *Br J Ind Med* **50**: 758–764

International Commission on Non-Ionizing Radiation Protection (ICNIRP) (1998) Guidelines for limiting exposure to time-varying electric, magnetic, and electromagnetic fields (up to 300 GHz). *Health Phys* **74**: 494–522

Kaatsch P, Kaletsch U, Meinert R and Michaelis J (1998) An extended study on childhood malignancies in the vicinity of German nuclear power plants. *Cancer Causes Control* **9**: 529–533

Knox EG and Gilman EA (1997) Hazard proximities of childhood cancers in Great Britain from 1953–80. *J Epidemiol Comm Health* **51**: 151–159

Linet MS, Hatch EE, Kleinerman RA, Robison LL, Kaune WT, Friedman DR, et al (1997) Residential exposure to magnetic fields and acute lymphoblastic leukemia in children. *N Engl J Med* **337**: 1–7

London SJ, Thomas DC, Bowman JD, Sobel E, Cheng T and Peters JM (1991) Exposure to residential electric and magnetic fields and risk of childhood leukemia. *Am J Epidemiol* **134**: 923–937

McBride ML, Gallagher RP, Theriault G, Armstrong BG, Tamaro S, Spinelli JJ, et al (1999) Power-frequency electric and magnetic fields and risk of childhood leukemia in Canada. *Am J Epidemiol* **149**: 831–842

Michaelis J, Schüz J, Meinert R, Zemann E, Grigat JP, Kaatsch P, Kaletsch U, Miesner A, Kalkner W and Kärner H (1998) Combined risk estimates for two German population-based case-control studies on residential magnetic fields and childhood acute leukemia. *Epidemiology* **9**: 92–94

Pfluger DH and Minder CE (1996) Effects of exposure to 16.7 Hz magnetic fields on urinary 6-hydroxymelatonin sulfate excretion of Swiss railway workers. *J Pineal Res* **21**: 91–100

Portier CJ and Wolfe MS (1998) National Institute of Environmental Health Sciences Working Group Report. Assessment of health effects from exposure to power-line frequency electric and magnetic fields. NIH publication no. 98–3981, Research Triangle Park: NIEHS

Reiter RJ (1997) Antioxidant actions of melatonin. *Adv Pharmacol* **38**: 103–117

Schüz J, Kaletsch U, Meinert R, Kaatsch P and Michaelis J (1999) Association of childhood leukaemia with factors related to the immune system. *Br J Cancer* **80**: 585–590

Schüz J, Grigat JP, Störmer B, Rippin G, Brinkmann K and Michaelis J (2000) Extremely-low-frequency magnetic fields in residences in Germany: distribution of measurements, comparison of two methods for assessing exposure, and predictors for the occurrence of magnetic fields above background level. *Radiat Environ Biophys* **39**: 233–240

Schüz J, Grigat JP, Brinkmann K and Michaelis J (2001) Residential magnetic fields as a risk factor for childhood acute leukaemia: results from a German population-based case-control study. *Int J Cancer* (in press)

Tynes T, Jynge H and Vistnes AI (1994) Leukemia and brain tumors in Norwegian railway workers, a nested case-control study. *Am J Epidemiol* **139**: 645–653

UK Childhood Cancer Study Investigators (1999) Exposure to power-frequency magnetic fields and the risk of childhood cancer. *Lancet* **354**: 1925–1931

© 2001 Cancer Research Campaign 

